# Cellular sociology regulates the hierarchical spatial patterning and organization of cells in organisms

**DOI:** 10.1098/rsob.200300

**Published:** 2020-12-16

**Authors:** Shambavi Ganesh, Beliz Utebay, Jeremy Heit, Ahmet F. Coskun

**Affiliations:** 1 Wallace H. Coulter Department of Biomedical Engineering, Georgia Institute of Technology and Emory University, Atlanta, GA, USA; 2 School of Electrical and Computer Engineering, Georgia Institute of Technology, Atlanta, GA, USA

**Keywords:** quantitative biology, cell-to-cell interactions, sociology of cells, multiplex bioimaging, spatial patterning

## Abstract

Advances in single-cell biotechnology have increasingly revealed interactions of cells with their surroundings, suggesting a cellular society at the microscale. Similarities between cells and humans across multiple hierarchical levels have quantitative inference potential for reaching insights about phenotypic interactions that lead to morphological forms across multiple scales of cellular organization, namely cells, tissues and organs. Here, the functional and structural comparisons between how cells and individuals fundamentally socialize to give rise to the spatial organization are investigated. Integrative experimental cell interaction assays and computational predictive methods shape the understanding of societal perspective in the determination of the cellular interactions that create spatially coordinated forms in biological systems. Emerging quantifiable models from a simpler biological microworld such as bacterial interactions and single-cell organisms are explored, providing a route to model spatio-temporal patterning of morphological structures in humans. This analogical reasoning framework sheds light on structural patterning principles as a result of biological interactions across the cellular scale and up.

## 1. Introduction

The proliferation of cells in the human body has intrigued the bioscience community and questions related to how cells are formed and how they communicate have been extensively explored in immunology, cancers and extracellular control mechanisms, among many others [1–5]. Strikingly, interactions at the cellular level are recapitulated in even complex human societies. As a cell is the basic unit of life, it has been widely studied for deciphering differentiation, specialization and life-death cycle. Similarly, humans are the basic units of society. To observe behaviour at the cellular level, multiple cellular profiling technologies have analysed gene expression, protein production and sequencing [6–9]. In parallel, large-scale human population data exhibit similarities in interaction at multiple levels of society that can be, for instance, studied by tracking behaviour patterns of humans in society using face recognition technologies [10], among others. These inferences from cell–cell or human–human interactions can better contribute towards unifying theories that link together intricacies of working principles in life formation, ranging from single cells to complex organisms including humans.

Cells respond to chemical signals in the environment around them as explained in the morphogenetic theory, in which morphological structures arise from differentiation and migration of cells that are dependent on the dissipation of biochemical signals, termed as morphogens [11]. Molecules carrying spatial guidance information in the embryo were found to be essential, which led to an appreciation of the importance of cellular interactions to construct a spatially patterned biological structure and organism [12]. Here, groups of cells working together form a unified cohort that contributes to the behaviour of a cell, as opposed to individualistic cellular decision making without the influence of others. Single cells lack certain properties (intracellular response state, cellular positions and extracellular messaging) that groups of cells acquire throughout their established communication [13,14]. A parallelism can be drawn between the paucity of acquired properties of cells withheld from group communication to that of insufficient characteristic maturation experienced by individuals devoid of any societal influence [15–17]. These surprising similarities between cellular and human interactions are covered in this comparative framework.

Herein, we investigate cellular interactions that lead to spatial forms in biology and, at the same time, provide analogies between the sociology of cells and human society. By looking at the similarities and dissimilarities, we reason that it is possible to study the complex ties underlying distinct micro- and macro-scale societies. To provide a framework emphasizing the interactions between cells, we explore the complexities in the spatial organization at cellular levels. Mechanisms similar in approach and formulation are pointed out as examples, gradually building up in the form of four hierarchical levels. When cell–cell relationships are investigated, the mechanisms involved in the communication within and between different cell groups are analysed. We then delve into spatial organization beyond that of the multi-cellular systems and consider organ spatial patterning and inter-organ communications.

## 2. Cellular spatial organization in a tissue

**Figure 1.**
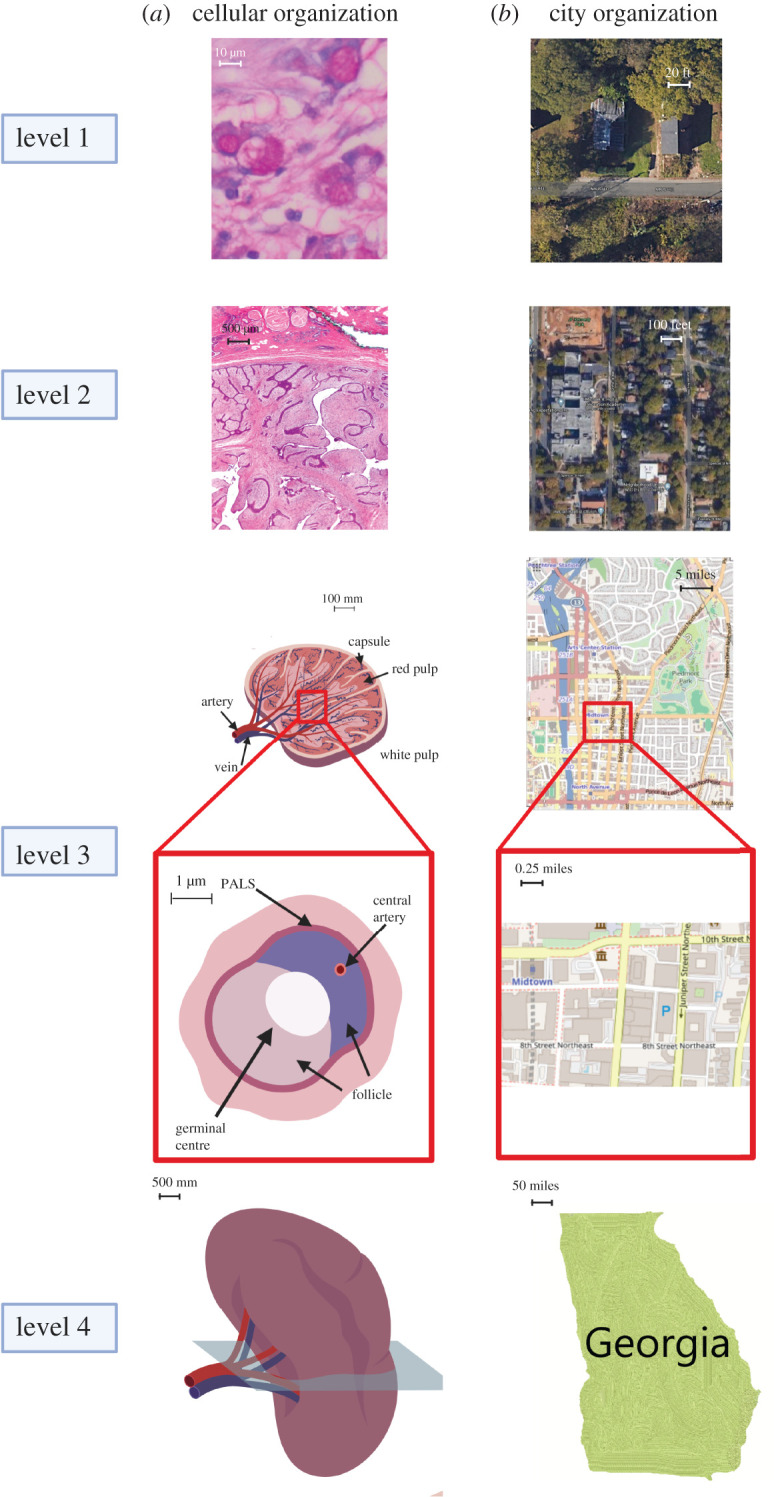
Hierarchical spatial regulation in cells and human cities. Comparisons between the structural similarities at the cellular level and the city level. (*a*) Hierarchical cell neighbourhoods: single cells reside in a tissue, stained by haematoxylin and eosin, at 10 µm scales (cellular level) and 500 µm scales (tissue level), a cross-section of the anatomical model (organ cross-section level) and the entire organ (organ level) are demonstrated. (*b*) City neighbourhoods: satellite image of city neighbourhoods in Atlanta by Google Maps at a 0.5-mile scale (building level) and 1-mile scale (neighbourhood level) are depicted. Districts in Atlanta at a 3 mile scale (suburb level) and the state of Georgia (city level) are presented.

This section expounds on the central idea that structural hierarchy implies functional properties at multiple scales. Structural hierarchy defines an organization containing multiple layers, in which every lower level is a subset of the higher levels of hierarchy. The depiction of the structural hierarchy is usually either through a pyramid chart or a tree diagram that progressively explores levels of increasing complexity from molecules to organisms, from the vantage point of a cell, and compares it to that of a human. Analogy starts with the base level of cells as a basic unit of life and humans as the fundamental element of society per the rubric put forward by society for population census [15]. Four hierarchical levels will be explored to cover the 10 µm scale (cellular level), a 500 µm scale (tissue level), a cross-section of an anatomical model (organ cross-section level) and a whole organ (organ level) (figure 1). In a nutshell, cells interact with each other to create tissues and organs, whereas human interactions construct city neighbourhoods, states and eventually countries.

Tissues are composed of a diverse variety of cell types. Multiple cellular phenotypes in a tissue cluster together in spatially distinct regions to perform a specific function. For example, the spleen is comprised the red pulp (i.e. filters the blood) and white pulp regions (i.e. regulates immune response with antibody production) as indicated by its anatomy (figure 1*a*) [16,17]. Structural and functional analogies can be drawn between the architecture of tissues and that of a city, whose structural organization covers business, residential and restaurant districts, among others (figure 1*b*). Similarly, biological information flows into and out of the tissue; here, veins and arteries are similar to the roads and highways that flow through and around a city [18]. Cities are divided into neighbourhoods because they share certain commonalities, such as the type of houses available and the cost of living [19]. For instance, the City of Atlanta is divided into distinct regions, such as downtown, midtown, Ponce and Old Fourth Ward based on the functional and structural role of individual regions in the mechanisms to form the city. The next higher level in the hierarchy corresponds to the State of Georgia that comprises multiple cities, while in the cellular case, it is the entire spleen organ.

**Figure 2.**
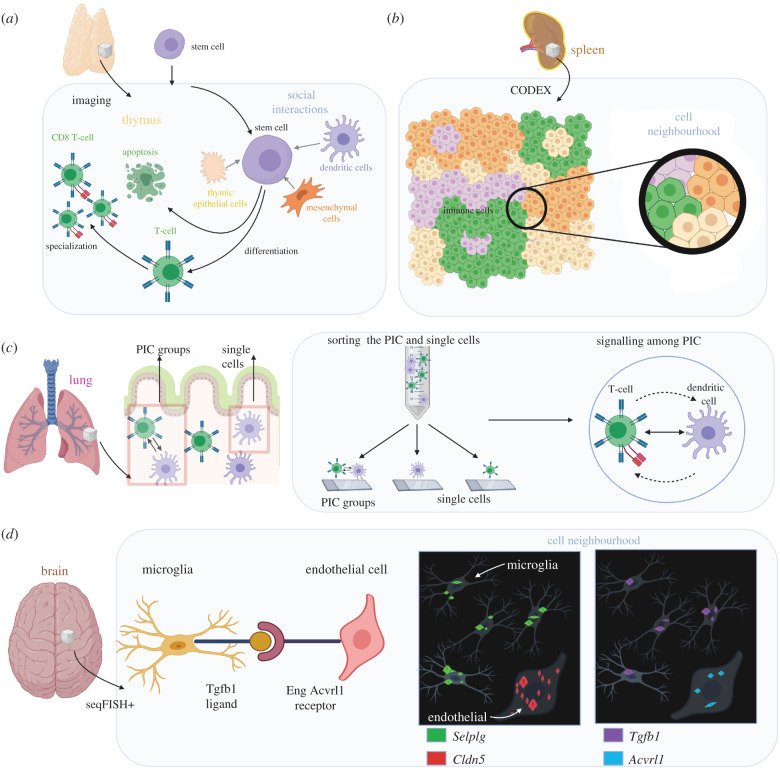
Cellular spatial organization in tissues. (*a*) Cells are subjected to internal, external and molecular interactions, contributing to the determination of cell fates. The haematopoietic stem cell differentiates into a CD8 T-cell with the help of cell types that comprise mesenchymal cells, thymic epithelial cells and dendritic cells. (*b*) Immune cell neighbourhoods were observed in tissues by multiplexing methods. One example is the repeated cell neighbours in a spleen that were observed and quantified through CODEX proteomic imaging. Unique cell types cluster together, leading to functional responses in single immune cells. (*c*) The PIC groups and the single-positive cell groups were identified in the neonatal murine lungs. The PIC cells were sorted and sequenced from the doublet cells. Finally, PIC-seq was used to map out interactions between T-cells and dendritic cells in the neonatal murine lungs. (*d*) The mRNA distributions in the cortex of a mouse brain were determined using seqFISH+ to reveal ligand–receptor interactions of microglia and endothelial cells.

Cells adopt a particular functionality that is moulded by external and internal influences, similar to humans deciding on taking up a particular direction in society. An example of external influences that contribute to the cells occurs in the thymus, wherein stem cells undergo differentiation and specialization into a variety of T cells under the influence of other cell types such as mesenchymal cells, thymic epithelial cells and dendritic cells surrounding them (figure 2*a*) [20]. Internal influences include the initial distribution of crucial compounds, such as transcription factors (e.g. those controlling T-cell fate determination using *Gata3*, *TCF-1* and *Bcl11b* genes), growth factors (e.g. Notch signalling) and other proteins, as well as corresponding gene expressions of the cell [21–25]. This concept can be compared to the decisions of humans in a society. The cellular decision is the role of the thymus, which has a key function in adaptive immunity.

The commonalities between haematopoietic cell maturation and decision making in human society lie in a myriad of influences that the haematopoietic stem cells and humans are subjected to reach the desired stage. Intriguing analogy occurs between the acquired collective memory of a group of cells and the way an individual acquires knowledge from his group from the inception of his life. Humans experience intricate influences that help them to choose a life path and career from the moment they are born under complex factors such as genes and external stimuli. Internal factors here are the personality of human beings, their inherent interests, ambition and skills, whereas the external factors are socioeconomic factors, culture, family, friends and situational experience acquired (figure 2*b*). Thus, human life cycles and cell cycles (i.e. leads to apoptosis) are highly parallel.

Recent advances in spatially resolved transcript and protein analysis can be directly applied to studying the interactions in the immune system. High-throughput RNA-imaging technologies, such as sequential fluorescence *in situ* hybridization (seqFISH) and multiplexed error-robust FISH (MERFISH) methods, can determine messenger RNA (mRNA) levels in single cells for up to 10 000 genes [9,26,27]. In MERFISH and seqFISH methods, the spatial RNA profiles that are extracted from tissue samples can then be modelled to dissect cellular communication using the cell identity from RNA distributions per cell and their proximity. High-content protein-imaging technologies, such as co-detection by indexing (CODEX) [28] and cyclic immunofluorescence [7], spatial metabolic analysis methods, including secondary ion beam mass spectrometry [29], matrix-assisted laser desorption/ionization mass spectrometry imaging [30] and other analysis techniques employing sequencing physically interacting cells (PIC-seq) [31] can be used to analyse molecular mechanisms of cellular communications for extracting their native interactions in tissues.

The methods to identify the different cell types in an organ through conventional imaging techniques such as histology and immunofluorescence are limited to the simultaneous profiling of more than five molecular signatures at a time. By contrast, the previously described CODEX technology can be used to capture the spatial organization of immune cells in organs because of its ability to detect more than fifty protein types simultaneously. Some autoimmune diseases may be associated with changes in the distributions of the immune cells within the tissue organization of the spleen [28]. The progression of such diseases can be detected through CODEX by observing cell neighbourhoods, micro-scale regions of tissue in which the central cell is repeatedly surrounded by a group of other cell types, in the spleen (figure 2*b*). Disruptions caused by tumours to the organization of immune cells in the neighbourhoods, such as exhaustion of T-cells, are linked to outcomes that are inferior in high-risk patients. This stems from interruptions to effective communication between cell neighbourhoods, which originally functioned as modules in the adaptive immune system. PIC-seq uses single-cell RNA sequencing to model cross-communications such as signalling in cell doublets that are typically ignored in classical flow-cytometry experiments [31]. PIC-seq identifies the PIC groups and single-positive cell groups that are sorted and then sequenced for characterization of the interactions between T-cells and dendritic cells in the neonatal murine lungs (figure 2*c*). An evolution of seqFISH (seqFISH+) is another imaging technique in spatial genomics that overcomes the problem of limited resolution. In seqFISH+, super-resolution imaging capabilities are achieved by the combination of standard commercial confocal imaging and sequential hybridizations. Using seqFISH+, mRNA over 10 000 genes were imaged in the cortex of a mouse brain to reveal ligand–receptor enrichment in neighbouring cells, in particular microglia and endothelial cells based on mRNA expressions (figure 2*d*) [26]. In the mouse brain, the pathways of ligand–receptor signalling were tissue-specific, highly localized concerning the positioning of nearby cells, and dependent on the expression of distinct mRNAs. Microglia were found to express transforming growth factor beta mRNA and endothelial cells near microglia expressed endoglin mRNAs.

Unique cell types contained in cell neighbourhoods of tissues can be partitioned by using segmentation algorithms that employ deep-learning and machine-learning concepts. Based on the cellular phenotypes and phenotypic interactions of the cells in the cellular neighbourhood, computational models can not only predict the growth and development of cells but can also segment the regions that contain different types of cells. Segmentation algorithms such as compression and histogram-based methods use deep-learning concepts based on convolutional neural networks (CNNs) that are preferred owing to scalability and accuracy of results [32]. For this purpose, a plethora of algorithms that use computer vision techniques such as watershed transformation, model-based segmentation and region growing methods can be used. The reason why deep learning is preferred is owing to the generalizability of the problem, given that the network receives the appropriate amount of training. This in turn minimizes error and maximizes accuracy [33–36]. Similarly, in cities, segmentation of districts using CNNs can be carried out. Distinct areas are characterized by permutations of factors such as businesses, entertainment centres and residential areas. Traffic regulation benefits from such data-driven segmentation of cities [37]. Based on quantitative metrics collected from the residents of a city, such as the reason for visiting the establishment and time spent in each establishment, the segmentation of these distinct areas is possible.

Cells interact with each other in their society. For example, intercellular communication of the immune system occurs both in the short and long-range. While short-range communication relies on cell–receptor interaction, long-range communication uses molecules such as tunnelling nanotubes (TNTs) and extracellular vesicles (EVs). TNTs are involved in cargo transportation during pathological changes that lead to diseases [38]. TNTs play critical roles in the survival of cells through the transportation of subcellular cargos after the onset of stress or injury [39]. EVs facilitate cancer growth based on the transport of mRNAs and proteins for long-range communication [40]. Secreted proteins are a fraction of the proteins produced by the cell and have a variety of functions, spanning defence, immunity and inflammation [41]. During an immune response, different signalling molecules work together to enhance or inhibit different aspects of immune cells such as their mobility, antibody production or phagocytosis. Whether or not a cell is responding to the signals can be measured with cellular profiling technologies. Mapping RNAs and proteins in cells can be used to dissect the interaction patterns among cells. For instance, proteomic signatures of 10 000 proteins in 28 human immune cell types obtained by mass spectrometry were used to map their social network [42]. These RNA sequencing and mass spectrometry techniques have generated extensive databases about cellular communications maps in many human systems, particularly in immunology. The significance of these social connections among cells is to regulate cellular decision making in response to intra-body signals.

## 3. Multi-cellularity in the spatial organization of organs

### 3.1. Organ patterning

**Figure 3.**
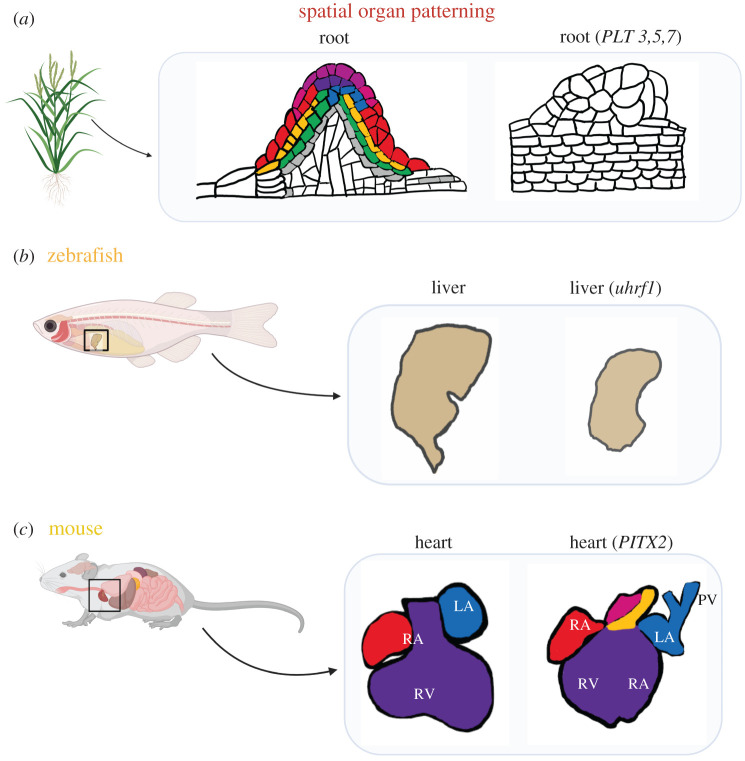
Multi-cellularity for the spatial patterning of organs. (*a*) The growth of a new axis is dependent on the activation of *PLT* *3*, *4* and *7* genes. Abnormal growth of roots in the plant arises from unbalanced cellular interactions. (*b*) Mutations of the *Uhrf1* gene impacted regeneration abilities and resulted in the malfunction of the liver in the various stages of development of a zebrafish. (*c*) In mice, mutations of the *PITX2* gene altered the cell composition and spatial symmetry of the murine heart.

Spatial patterns of organs in an organism are regulated by the sociological interactions of their cellular constituents, which are mediated through key protein factors [43]. At the same time, organ pattern formation is regulated by modifications in gene expression [44–46]. Spatial formation rules of organs are evolutionarily conserved, and thus, numerous organisms of plants, flies and fishes were analysed for their intra-organ structures. In plants, the growth of meristems was initiated and organs were produced based on the effects of *PLETHORA* (*PLT*) activation factors [47]. The expression of various *PLT* genes contributed to the identities that were acquired by the cells during division, causing them to alter the formation of a new growth axis or a new organ (figure 3*a*). Such sociological factors contribute to spatial organ patterning in plants.

In complex vertebrates, cellular regulation is often modified by sociological drivers such as gene switches that alter the communication channels of cells. Zebrafish are commonly studied in the context of tissue damage and regenerative capabilities [48]. Mutations of the *Uhrf1* gene that are responsible for hepatic outgrowth impacted the embryos of zebrafish through cell cycle modifications [49], resulting in abnormal liver growth in the various stages of development of the zebrafish (figure 3*b*). In this spatial model, the liver growth was studied in particular during the hepatogenesis stage of embryonic development and the regeneration stage after partial hepatectomy of the zebrafish for organ patterning.

Endoderm cells in mice drive spatial patterning from the progenitor tissues within specified organs at an early stage, as a small fraction of the cells were found to have acquired localization-based traits during early stages of development that later could be matched to a sequence of organ identities [50]. During embryogenesis, endoderm cells acquire an identity that pre-defines their spatial positioning [51,52]. For instance, the cell composition and symmetry of the murine heart were altered by mutating the *PITX2* gene (figure 3*c*), wherein the *PITX2* gene also affected the development of the heart valves [53]. These conserved findings in plants and animals showed that any modifications in the communication channels of the cells would cause morphological changes in the spatial patterning of distinct organs.

### 3.2. Organ positioning in humans

Organs in a human body also exhibit location preference based on cellular society. For instance, malformations and positional displacement of organs owing to birth defects, often accompanied by congenital heart defects, is known as a multi-organ disorder called heterotaxy [54]. In this syndrome, mutations of certain genes such as *CRYPTIC*, *LEFTYA*, *ZIC3*, *NKX2.5* and *ACVR2B* are observed to contribute towards human heterotaxy, specifically disturbing left-right patterning in the early stages of embryonic development. This disruption to left-right patterning manifests itself in the form of segmental discordances and atrial isomerism of the thoracic and abdominal positioning of organs in the body [55,56]. Intestinal rotation abnormalities have been commonly observed in infants found to have heterotaxy syndrome and expressed symptoms by the age of six [57]. Abnormal structures of the chest and abdominal organs are common in children with heterotaxy syndrome [58]. Mutations of genes associated with the Bardet–Biedl syndrome genes lead to ciliopathies, which are in turn associated with changes in the placement and asymmetry of organs [59,60]. Therefore, humans with heterotaxy syndrome exhibit a range of phenotypic variability, the complete identification of which is essential for comprehensive analysis of genetic etiology. On the other hand, while genomic tools to determine the spatial patterning of human organs have been limited, the anatomy of humans can be modelled based on probabilistic atlases from registered computed tomography scans.

## 4. Scalable spatial organization from cells to humans

Multiple models can be used to quantify the sociable actions exhibited by cells. There has been increasing interest in describing the growth of tumours, bacterial colonies and single cells [61,62] to investigate cancer therapy, microbial population dynamics and *in vivo* imaging of organs. Mathematical modelling has been powerful as a tool for interpreting cellular and molecular mechanisms and has been useful in modelling the behaviour of several higher level organisms.

In cell societies, various parameters such as cell cycle, proliferation and motility are commonly studied as a function of time. The environment of a cell can controllably be adjusted to study chemical gradients of stimulants that completely alter the growth trajectory of the cell. With this systematic approach, mathematical models have been developed such as reaction–diffusion models, evolutionary game theory models and numerical modelling [63–65]. These computational models can then be fine-tuned based on the simulations, outputs and parameters, providing ample opportunities to decipher the spatial dynamics that use the concept of cellular sociology. Mathematical models have been used to interpret the behaviour of higher-order organisms. For instance, frameworks to analyse collective manners in harvester ants colonies using collision theory and random walk models reveal trends in patterns of interaction between ants [66]. The integration of behavioural models using partitioning algorithms describes the social practices in the swarms of bees and ant colonies [67]. Additional models simulate the human responses for epidemic scenarios during evacuations in the case of emergencies [68–71].

Cell movement has extensively been modelled by mathematical foundations [72,73]. When a chemical gradient is formed in an environment, migration models are often used to predict the relocation patterns of a cell. Several chemotaxis models have been used to predict the movement of population densities [74]. Mathematical models of migration have been created to quantize these results. The factors that influence the drifting of single-celled organisms are the concentration and direction of chemotaxis, positioning of similar organisms and threat of predators. The migration of animals has been analysed extensively using mathematical models such as periodic Markov models and stochastic optimal stopping formalism theory [75,76]. To quantify the cohesive behaviour of insects, stochastic compartmental models have been investigated. Similarities and differences in collective migration are studied across organisms of varying complexities using mathematical models, giving rise to a unifying theory on collective migration from simple cells to complex organisms [77].

The international migration of humans globally has been affected by logistics, cultural, and geographical distances. Population patterns among countries exhibit preferential connectivity [78,79]. The associated spatio-temporal patterns have been investigated using complex network theory to study the correlation between international human migration and its influence on bilateral trade between countries [80,81]. Modelling these relationships provides insight into the mobility patterns of humans in the prediction and control of diseases [82].

## 5. Conclusion

In this commentary, we examined the cellular interactions underlying the hierarchical spatial organization of tissues and organs. Environmental factors such as signalling and internal influences comprise molecular networks that drive the sociology of cells, leading to intra-organ spatial patterning rules. Multiplex and high-content imaging technologies have opened the doors to probe cellular associations based on physical interacting cell-pairs, ligand–receptor relationships and spatially regulated gene regulation analysis in nearby cells. Sociological switches regulate cellular decision making to determine the eventual spatial organization of organs in plants and animals. The phenotypic variability that is involved in the genetic syndromes contributes to the positioning of organs in humans. Mathematical models have been instrumental in the integrated analysis of behaviours in cells and higher-order organisms. With the advancement in single-cell biotechnologies and cell–cell interaction modelling, the cellular sociology framework paves the way for universal principles of spatial patterning from individual cells to organs.

## Data accessibility

This article has no additional data.
